# Facemasks Block Lower Visual Field in Youth Ice Hockey

**DOI:** 10.3389/fspor.2021.787182

**Published:** 2021-12-06

**Authors:** Kyle Critelli, Victoria Demiris, Brooke N. Klatt, Benjamin Crane, Eric R. Anson

**Affiliations:** ^1^Department of Otolaryngology, University of Rochester, Rochester, NY, United States; ^2^Physical Therapy Department, University of Rochester, Rochester, NY, United States; ^3^Physical Therapy Department, University of Pittsburgh, Pittsburgh, PA, United States; ^4^Department of Neuroscience, University of Rochester, Rochester, NY, United States; ^5^Department of Biomedical Engineering, University of Rochester, Rochester, NY, United States

**Keywords:** facemask, ice hockey, safety, COVID-19, visual fields

## Abstract

Wearing a facemask (FM) reduces the spread of COVID-19, but it also blocks a person's lower visual field. Many new public safety rules were created in response to COVID-19, including mandated FM wearing in some youth sports like youth ice hockey. We hypothesized that FM wearing in youth hockey players obstructs the lower field of view and may impact safety. Youth hockey players (*n* = 33) aged 12.03 (1.6) years button press when they saw an LED on the floor turn on in two conditions (wearing FM or no FM) in random order. An interleaved one-up/one-down two-alternative-forced-choice adaptive staircase design was used. Visual thresholds were calculated for each condition and participant. The visual angle threshold (VAT) was determined using standing eye height and the linear distance from the tip of the skates to the visual threshold. Paired *t*-tests determined whether mask wearing changed the VAT. We modeled the probability a player could see the puck on their stick in four distinct scenarios to estimate the potential impact of FM wearing during hockey play. The average unmasked VAT (11.4 degrees) was significantly closer to the skates than the masked VAT (20.3 degrees) (*p* < 0.001). Our model indicated a significant reduction in ability to visualize the puck using peripheral vision when more upright while wearing a FM. FM wearing compromised their lower visual field, suggesting a downward head tilt may be necessary to see the puck. Playing ice hockey while wearing a FM may lead to unsafe on-ice playing conditions due to downward head tilt to see the puck.

## Introduction

While the COVID-19 pandemic is far from eradicated, there are definite signs of hope for a return to normalcy. Vaccine distribution has begun and most adults and teenagers (but not children under 12) are eligible to receive the vaccine in the US (Centers for Disease Control Prevention, 2021). Several studies have demonstrated that policies of universal masking contribute to preventing the spread of COVID-19 (Hendrix et al., 2020; Payne et al., 2020; Van Dyke et al., 2020; Wang et al., 2020), and the Centers for Disease Control and Prevention continues to recommend mask wearing for non-vaccinated individuals (Centers for Disease Control and Prevention) and many institutions continue to have mask mandates, especially for non-vaccinated individuals. With the emergence of contagious variants, considerations other than disease transmission are relevant to decisions about universal masking in youth sports. This is highly relevant as winter approaches and there is a greater emphasis on indoor sports that were largely cancelled or required athletes to wear masks during play (Dergaa et al., 2021).

Although wearing a facemask mitigates the spread of COVID-19, wearing a facemask has also been shown to block the lower visual field (El-Nimri et al., 2020; Young et al., 2020; Klatt and Anson, 2021; Weber et al., 2021). Restricting the peripheral visual field increased reaction times and has been linked to reduced overall sports performance in football and downhill skiing (Loopeker and Rowley, 2001; Ruedl et al., 2011; Miller et al., 2019). Blocking the lower visual field promotes downward head tilt, which reduces how far ahead obstacles can be detected (Timmis and Buckley, 2012). Blocking the lower visual field during walking may lead to a trip or fall. Obstruction of the lower visual field related to wearing a facemask may worsen balance for people with higher fall risk (Kal et al., 2020; Klatt and Anson, 2021). Another arena where facemask wearing may have detrimental consequences related to increased risk for injury is the sport of ice hockey. Ice hockey was designated as a “moderate or high-risk” sport related to the potential for COVID-19 spread in several US states, and rules were developed for players/coaches/officials to wear facemasks (Maine Department of Economic Comminuty Development, 2021; MinnesotaHockey, 2021). In some cases, wearing facemasks was mandated for any on-ice hockey activities at all age levels. However, not all states/countries adopted this mandate for players. Although most early evidence suggests that healthy individuals are only mildly impacted while wearing a FM during exercise (Shaw et al., 2020; Hopkins et al., 2021; Mapelli et al., 2021; Shein et al., 2021), others have reported significant reductions in respiratory capacity in healthy adults (Fikenzer et al., 2020; Lässing et al., 2020; Driver et al., 2021). To date the impact of mask wearing on aspects of exercise or sports participation, such as player safety, has received little attention.

Ice hockey players wearing a FM may have reduced lower field of view and to facilitate seeing the puck may need to tilt their head down. Tilting the head downward reduces the “look ahead” window, effectively shortening the reaction time for contact avoidance (Patla and Vickers, 2003). In fast moving contact sports like ice hockey, not being able to see your opponent soon enough because of downward head tilt may lead to more frequent body contact/checking which is the most common cause of player injury (Brust et al., 1992; Emery et al., 2011; Brenner et al., 2014; Mosenthal et al., 2017; Simmons et al., 2017). The Hockey Equipment Certification Counsel Inc. promotes an education program titled “Heads-Up, Don't Duck” to specifically create awareness of the increased risk of cervical spine injuries when looking downward (HECC). Therefore, a scenario where ice hockey players tilt their head downward to play the puck due to a restricted lower visual field (from wearing a facemask while playing), may lead to unsafe on-ice conditions.

Although it has long been known that blocking the lower visual field impairs walking ability (Marigold et al., 2007; Marigold and Patla, 2008) and reaction times in football and alpine skiing (Ruedl et al., 2011; Miller et al., 2019), the extent to which wearing a mask blocks the lower field of view in the context of ice hockey is unknown. Here we report on whether mask wearing in youth hockey players significantly restricts their lower field of view. We hypothesized that youth ice hockey players would experience a significant reduction in the lower visual field when wearing a facemask. We additionally modeled the relationship between the FM induced reduction in the lower visual field and the ability to see a puck on their stick. We hypothesized that wearing a facemask would impair the ability of youth hockey players to use peripheral vision to see a puck on their stick.

## Methods

### Subjects

Thirty-three youth hockey players (5 girls, 28 boys) average age 12.03 (SD 1.6) were recruited and provided written assent (ages <18) to participate in this study approved by the institutional review board at the University of Rochester Medical Center. Written parental permission was also provided for participants under 18. All experimental participation took place at Rochester Ice Center in Fairport, NY. At the time of data collection, all persons inside the building at Rochester Ice Center were required to wear masks, except when on the ice for practice or games. Participants were tested using the FM they wore at the time of testing as this method pragmatically represented the FM type they were likely to wear when playing. No effort was made to adjust fit of the FM for any participant. Participants were compensated with a $15 gift card to a sporting goods store after completion.

### Experimental Setup

#### Apparatus

**Figure 1:**
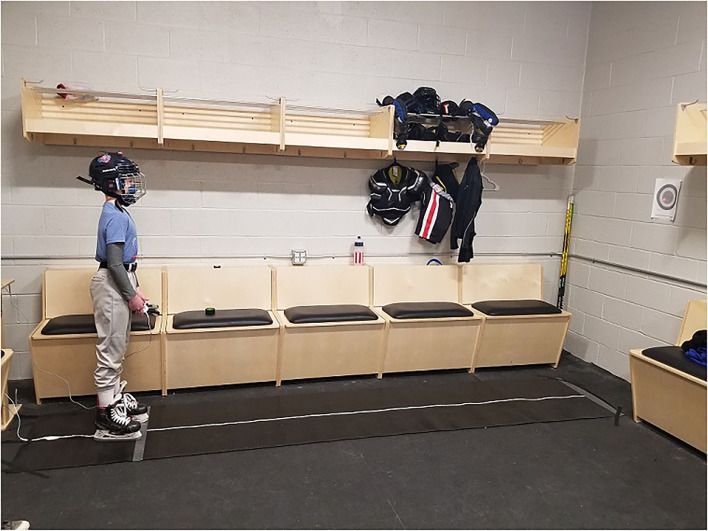
An exemplar demonstrating the experimental set-up. Participants were instructed to keep the helmet mounted laser pointer in the center of the bulls-eye (Radius 9.1 cm) taped to the wall at eye height 12 feet away. Subjects stood with a comfortable stance straddling the LED strip in their skates holding the Xbox controller so that it was not visible while looking straight ahead.

An individually addressable light emitting diode (LED) strip (WS2812B ECO LED Strip, BTF-Lighting Technology Co., Ltd., Guangdong, China) three meters in length (180 LEDs, 60 LEDs/m) was centered on a black plastic mat, see Figure 1. Distance between each LED was 12 mm. During the experiment, individually addressed LEDs were powered for 200 ms and specified to be white light for maximum contrast with the black plastic mat. These colors were selected to better simulate the color contrast of the typical black hockey puck on a white ice surface. A wired Xbox (Microsoft, Inc., Redmond, WA) controller allowed participants to respond during the protocol by clicking either the right or left buttons with their index fingers for “yes” and “no” responses, respectively. A custom MATLAB 2018a (The MathWorks, Inc., Natick, MA) script and an Arduino Nano3 (Arduino LLC., Torino, Italy) were used to play the auditory tone and turn on/off individual LEDs as well as capture participant responses (Xbox button press). All data were analyzed offline using custom MATLAB scripts.

#### Protocol

After providing assent/permission or consent to participate, each participant provided demographic data including age and sex, and sport specific information including years played, level of play (house or travel), whether they wear glasses during play, type of faceguard (wire/clear plastic), their playing position (center/wing/defense/goalie), and the type of personal facemask they wore for the experiment, see Table 1 for demographics. Participants indicated whether they had experienced a concussion or other injuries in the last 6 months during play. Participants indicated whether they wore a facemask while skating during either practice or games. If they responded “yes” to either of those questions they were asked what type of mask they wore and whether they were required to wear a mask. If required to wear a mask they indicated who provided the mask for them to use. Finally, participants who reported wearing a mask during practice or game play were asked, “Do you feel you have to look down more with the mask on to see the puck than when you do not wear a mask?” While wearing their hockey skates standing height, standing eye height, hockey stance eye height, and distance from the tip of their skates to the toe and heel of their stick were measured for all players except goalies. Hockey stance positioning includes holding the stick with both hands, resting the stick blade on the floor in front of them with their head up, back straight, and both knees slightly bent. This terminology is common in youth hockey all participants easily understood.

**Table 1:** Participant demographics.

**Participant demographics**	
Age	12.03 (1.6)
Sex	
Male	28
Female	5
Glasses	1
Years played	6.7 (1.89)
Hockey level	
House	18
Travel	15
Helmet face guard type	
Wire	28
Clear	5
Mask type during testing	
Cloth	26
Surgical	6
Gator	1
Position	
Forward	18
Defense	11
Goalie	4
Injuries	
Concussion	1
Other	3
Wear a mask in practice (yes)	5 (15.1%)
Wear a mask in games (yes)	9 (27.3%)
Standing height in skates (cm)	160.21 (11.32)
Standing eye height in skates (cm)	148.67 (11.08)
Hockey stance eye height in skates (cm)	122.94 (12.50)
Skates to stick blade (cm)	59.21 (27.58)

A laser pointer was attached to their helmet and participants were then familiarized with the two-alternative-forced-choice (2AFC) paradigm with a demonstration that included 10 auditory cues (300 ms 200 Hz tones) that served as a prompt to respond. For six auditory cues an LED turned on, and for four auditory cues no LED was turned on. During the familiarization task, participants were allowed to look directly at the LED strip to ensure visualization of the LEDs when turned on. This task familiarization ensured that the participants would click the correct response button. The familiarization task was repeated until the participant demonstrated understanding of the task. The experimental set up is shown in Figure 1.

Participants were instructed to keep the helmet mounted laser pointer “as close to the center of a bulls-eye target as possible” which was taped to the wall at their eye height (in skates) 12 feet in front of them. This distance was determined to be achievable but sufficiently challenging to require continuous visual attention in early piloting. Participants were tested in two conditions (wearing a facemask or no facemask) in random order determined by a coin flip. Each condition consisted of 60 trials (paired audio cues and responses). An interleaved 2AFC adaptive staircase design was used with 30 trials per staircase (Levitt, 1971). Each staircase included five trials with auditory cues intentionally paired with the LED remaining off to check for task attention (trials 3, 9, 17, 21, 25 for the descending staircase and 2, 10, 16, 22, 27 for the ascending staircase). The remaining 25 cues presented synchronous auditory and visual (LED) cues. The auditory cues were 300 ms 200 Hz tones from the internal speakers of the data acquisition computer at maximum volume (Dell Lattitude 7400, Dell, Inc.). The visual cues were white light LEDs turned on for a 200 ms duration. This supra-threshold stimulus duration was twice that needed for a visual discrimination task (Croker and Maratos, 2011). We opted for a brief but supra-threshold visual stimulus as the hockey puck is never invisible. The inter-trial interval varied randomly between 1.5 and 2.5 seconds.

For both staircases, the maximum LED number was 182 and the minimum LED number was one. Whether a trial was to test the up or down staircase was determined randomly, with two exceptions. The number of consecutive steps per staircase was randomly determined (integers between one and five) following each staircase reversal. The next step(s) must be for the other staircase. Additionally, if all 30 trials were completed for a particular staircase, the remaining trials must all be from the other staircase. The step sizes were predefined in order [20, 16, 14, 12, 10, 9, 7, 5, 3, 2, 1] allowing for 11 staircase reversals or consecutive changes in step sizes (Wetherill and Levitt, 1965). This method ensures more stimuli near the threshold (Roditi and Crane, 2012). If the next step size was less than the pre-set minimum step size (one LED), the step size was specified as one LED.

### Data Analysis

As a first pass, the ascending and descending staircases were examined for convergence based on overlapping histograms to ensure appropriate task performance. The binary data (no = 0, yes = 1) were bootstrapped with 100 replications and fit to a psychometric curve. The mean of the bootstrapped psychometric fits was calculated as the threshold (in units of LEDs) for each condition and participant and corresponded to the 50% point of subjective equality (Levitt, 1971; Roditi and Crane, 2012). The visual angle threshold was determined using standing eye height and the linear distance from the tip of the skates to the LED threshold. A paired *t*-test determined whether mask wearing changed the visual angle threshold. Hockey stance visual angle was determined using hockey stance eye height and the midpoint of the stick blade for all non-goalie players. To estimate the potential impact of mask wearing during hockey play, we modeled the probability a player would see the puck in several simulated conditions using Solver (Frontline Systems, Inc.). We created four normally distributed simulation models bootstrapped on the collected empirical data (mean and standard deviation). We ran 10,000 replications for each simulation. The four models used the visual angle threshold with mask and without mask as well as the calculated visual angle from eye height of the subject standing upright and in hockey stance with respect to the distance between the skates and the middle of the hockey stick. The models were run with logic, pairing conditions together to resemble four distinct possible outcomes simulating hockey playing: (1) masked paired with upright height (least ideal scenario), (2) unmasked paired with hockey stance height (most ideal scenario), (3) unmasked paired with upright height, and (4) masked paired with hockey stance height. Alpha was specified for each statistical comparison as α = 0.05 as they were considered independent questions.

## Results

Four participants did not demonstrate staircase convergence (indicating incorrect task performance) and one participant had missing data due to technical problems and were not included in the statistical analyses. The participants were 84.8% male and 45.4% (*n* = 15) played travel hockey last season, with the remaining participants being house players. Four individuals reported an injury last season, and nine individuals reported wearing a mask during practice or game play at least for some of the season, see Table 1. Six of the nine individuals reported that they had to look down to play the puck when wearing a mask compared to when unmasked.

**Figure 2:**
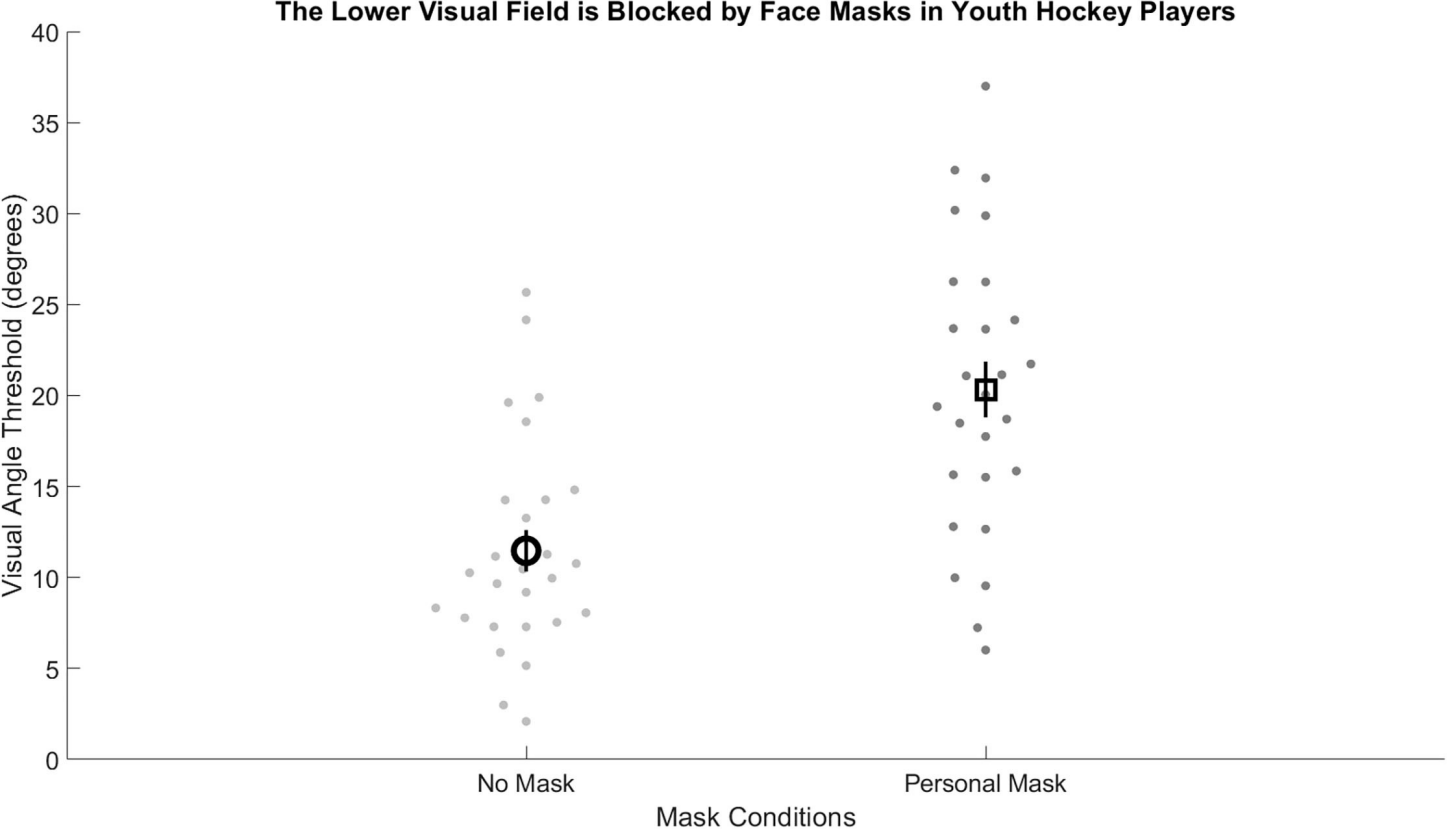
Visual angle thresholds for the no mask (light gray) were significantly smaller than for the masked (dark gray) condition. Group averages represented by a bolded circle (no mask) and a bolded square (masked). Error bars represent standard error.

The average unmasked visual angle threshold [11.4 degrees (SD 5.9 degrees)] was significantly smaller (closer to the skates) than the masked visual angle threshold [20.3 degrees (SD 8.0 degrees)], [*t*(1, 26) = −6.2515, *p* < 0.0000012, Cohen's *d* = −1.2], as shown in Figure 2. The average hockey stance visual angle was 29.73 degrees (SD 3.79 degrees). Four of the players had visual thresholds that exceeded their hockey stance visual angle.

### Model Results

**Figure 3:**
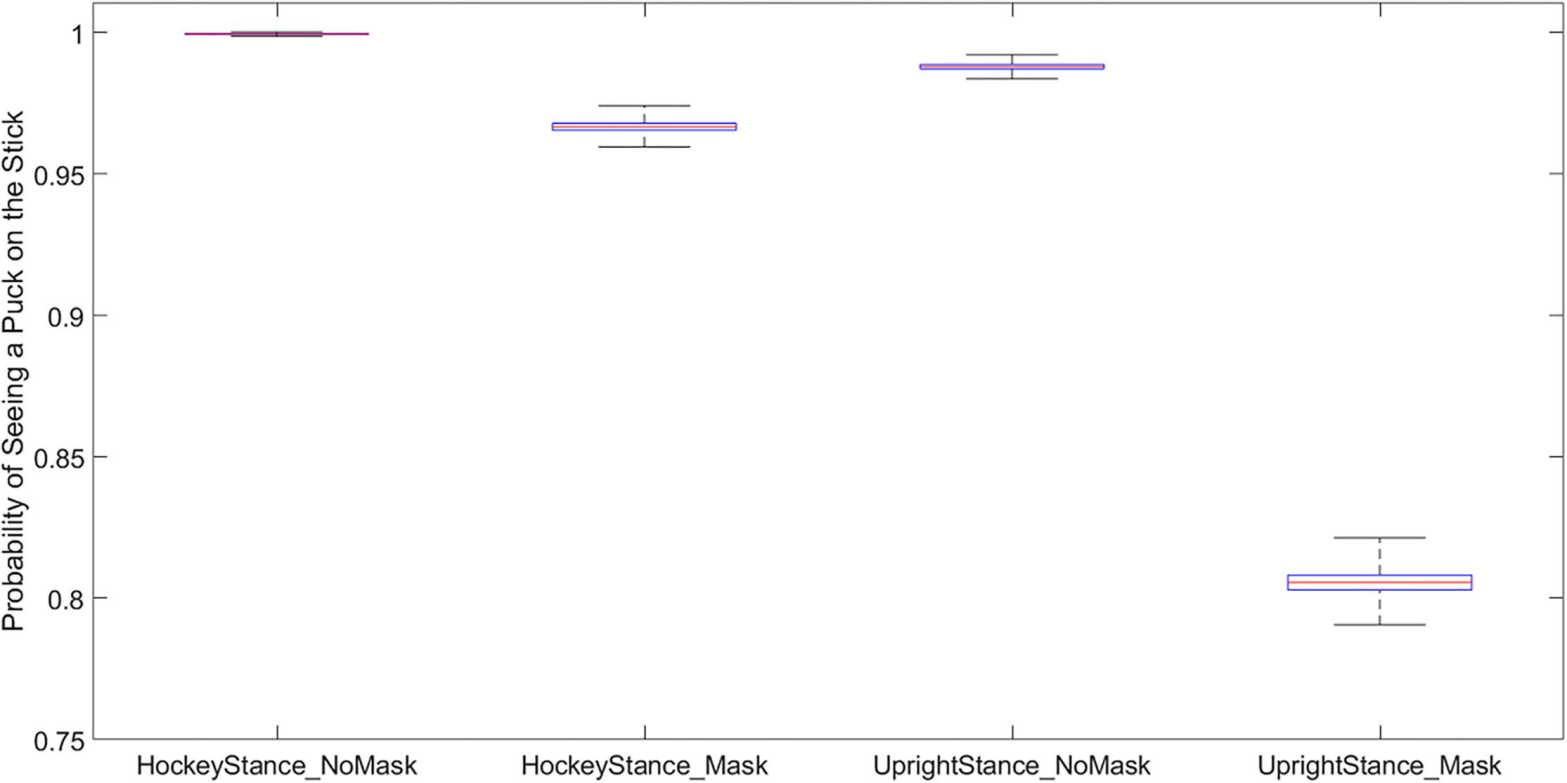
A box plot depicting the cross model results. This plot shows the percent chance the player could see the center of the stick blade compared across various conditions. The y-axis is the probability that the puck will be seen (yes = 1), note the significant drop in puck on stick visibility (~20%) when masked players are more upright.

**Figure 4:**
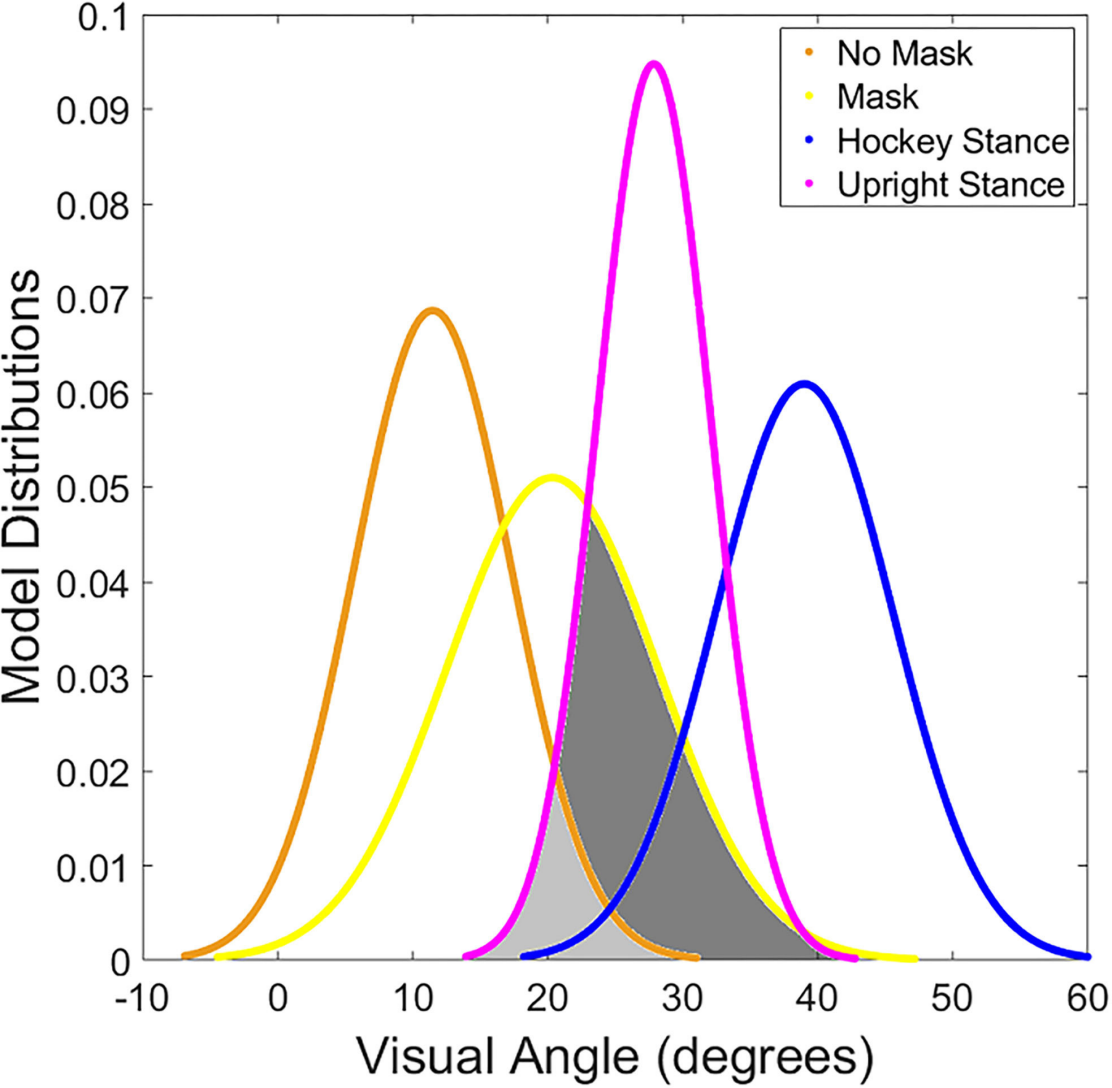
Depicts the distribution of visual thresholds determined for each model. The yellow (FM condition) and orange (No mask) lines illustrate the range of visual angles based on the empirical visual angle threshold data. The purple (upright stance) and blue (hockey stance) lines show the range of visual angles for the cohort across the height conditions. Where the orange or yellow line overlaps the blue or purple line (shaded in light and dark gray for emphasis) represents circumstances where the puck on the stick would not be visible without tilting the head down.

The results of the Solver models indicated approximately 20% reduction in the probability that a player would be able to see a puck on their stick blade using peripheral vision if wearing a facemask and standing closer to upright, see Figure 3. A further comparison of the distribution of visual angle thresholds compared to visual angles necessary to see the center of the hockey stick blade demonstrated significant overlap (overlap of 90% confidence intervals) between empirically derived masked visual angle thresholds and estimated visual angles needed in both hockey stance and upright stance conditions, see Figure 4. The model results demonstrate that players will periodically need to tip their head down to visualize the puck while skating.

## Discussion

The primary result from this novel investigation indicates that wearing a facemask significantly restricts the lower field of view in youth hockey players. Although not surprising, to our knowledge this has not previously been demonstrated in youth hockey players. These thresholds suggest that in a “heads-up” hockey stance, the lower visual field angular thresholds would allow most if not all tested players to visualize their stick blade only when not wearing a mask. When wearing a facemask, the fraction of time that the puck would be visible in the peripheral field of view is significantly reduced. Four of the non-goalie participants had a masked visual angle threshold larger than the visual angle from the eyes to the middle of the stick blade while in a hockey stance. Those four individuals would not be able to use peripheral vision to see the puck on the stick blade while skating with a mask on. Thus, to visualize the puck on the stick players must tilt their head down rather than relying on peripheral vision.

We further examined the potential for on ice change in head tilt behavior by relating the masked visual angle threshold to the calculated visual angles in both a hockey stance and an upright stance. It is unlikely that players maintain the exact hockey position height while in play on the ice. Our model conservatively accounts for this by examining the frequency the puck would not be visible given masking and stance criteria while holding the stick placement relative to the skates constant. While masked we expect the players to be able to see the puck using peripheral vision roughly 96% while in their hockey stance. The probability of seeing the puck when more upright is reduced to about 80%. Often during game play players skate in a more upright position than the hockey stance which is more analogous to the non-skating face off position. An important caveat to the interpretation of these models is that if the stick is not perfectly straight out in front of a masked player, the masked player may have to look down more often as the visual angle will shift closer to the visual threshold. Similarly, if the puck was held to the player's side the distance between puck and player would be shortened, further reducing visibility. Although we employed a conservative model to more closely link the visual angle thresholds to skating performance, additional studies are needed to examine on ice performance including both maintaining puck possession and avoiding body contact while masked.

Overall, the combined visual threshold results and modeling suggest that youth hockey players wearing facemasks would need to adopt head-down behaviors more frequently to see the puck. Head-down positioning increases the chance for player-to-player collision which is the primary cause of on-ice injury (Brenner et al., 2014; Black et al., 2016; Simmons et al., 2017). Restricted peripheral vision has been shown to reduce reaction times for visual targets detection (Miller et al., 2019). Hockey players with only slightly obstructed visual fields from protective equipment (face cages and face shields) also demonstrate slower reaction times to visual targets (Ing et al., 2002; Dowler et al., 2009). Increased oculomotor variability for target tracking and gaze shifting was associated with more frequent high acceleration collision impacts in youth ice hockey (Kiefer et al., 2018). The current results suggest that the on-ice use of FM in youth hockey may increase the risk for on-ice injury. The current results do not address potential for disease transmission during play, but highlight a previously unknown risk to player safety that has relevance to universal masking *during participation* in youth ice hockey. Ice hockey players appear to be special case population such that facemask wearing during participation may have unintentional effects that increase risk for injury.

## Limitations

The main limitation to this study is the off-ice, non-play oriented determination of a lower visual field threshold. The LEDs represent a stationary target, and thus a player's ability to intercept a moving puck cannot be determined. However, skating while possessing the puck may require at least periodic visual updating. We employed a conservative model to account for the static nature of the testing environment. Despite being well representative of the participants in this study, variations in facemask styles may not represent those used by players not participating in this study. Older youth hockey players or adults may have different results. This study did not address the impact of mask wearing during ice hockey play on disease transmission or respiration. The small sample size prevented within group comparisons to determine effects from position or type of face shield.

## Conclusion

Facemask wearing restricted the lower visual field in youth hockey players. Puck on stick visibility was compromised suggesting an increase in downward head tilt would be necessary to see the puck on the stick while playing ice hockey while wearing a FM. The current results suggest that the on-ice use of FM in youth hockey may be associated with increased use of head-down positioning that might lead to greater injury incidence during play. However, the association between FM use and an increase in injury risk should be evaluated directly.

## Data Availability Statement

The raw data supporting the conclusions of this article will be made available by the authors, without undue reservation.

## Ethics Statement

The studies involving human participants were reviewed and approved by University of Rochester, Research Subjects Review Board. Written informed consent to participate in this study was provided by the participants' legal guardian/next of kin. Written informed consent was obtained from the minor(s)' legal guardian/next of kin for the publication of any potentially identifiable images or data included in this article.

## Author Contributions

EA and BK conceived and designed the study. EA, KC, and VD collected study data. EA, KC, and BC performed data analysis. EA drafted the initial version of the manuscript. EA, VD, KC, BK, and BC critically edited the manuscript. All authors contributed to the article and approved the submitted version.

## Funding

EA was supported in part by the National Institutes of Health (NIDCD K23 DC018303).

## Conflict of Interest

The authors declare that the research was conducted in the absence of any commercial or financial relationships that could be construed as a potential conflict of interest.

## Publisher's Note

All claims expressed in this article are solely those of the authors and do not necessarily represent those of their affiliated organizations, or those of the publisher, the editors and the reviewers. Any product that may be evaluated in this article, or claim that may be made by its manufacturer, is not guaranteed or endorsed by the publisher.
